# Abnormal Hippocampal BDNF and miR-16 Expression Is Associated with Depression-Like Behaviors Induced by Stress during Early Life

**DOI:** 10.1371/journal.pone.0046921

**Published:** 2012-10-08

**Authors:** Mei Bai, Xiongzhao Zhu, Yi Zhang, Sheng Zhang, Li Zhang, Liang Xue, Jinyao Yi, Shuqiao Yao, Xiuwu Zhang

**Affiliations:** 1 Medical Psychological Institute, Second Xiangya Hospital, Central South University, Changsha, Hunan, China; 2 Departments of Radiation Oncology, Duke University Medical Center, Durham, North Carolina, United States of America; Radboud University, The Netherlands

## Abstract

Some environmental stressors lead to the onset of depression via inhibiting hippocampal BDNF expression, but other environmental stressors-induced depression exhibits no change in BDNF expression. The underlying mechanisms behind the divergence remain unknown. In this study, depression-like behaviors were induced in rats by maternal deprivation (MD) and chronic unpredictable stress (CUPS). Depression-like behaviors were tested by open field test, forced swimming test, and sucrose consumption test. BDNF and miR-16 expressions in the hippocampus were examined by real-time PCR. MD and CUPS rats crawled less distance, exhibited decreased vertical activity, and produced more fecal pellets than control rats in the open field test. However, MD rats crawled less distance and produced significantly less fecal pellets than CUPS rats. In the forced swimming and sucrose consumption tests, CUPS and MD rats exhibited longer floating time and consumed less sucrose than control rats, but MD rats exhibited shorter floating time and consumed less sucrose than CUPS rats. MD but not CUPS rats showed lower BDNF mRNA and higher miR-16 expression than control rats. In MD rats, BDNF mRNA expression negatively correlated with the expression of miR-16. BDNF expression positively correlated with the total distance rats crawled and vertical activity in the open field test while miR-16 expression negatively correlated the two behaviors. BDNF positively correlated with sucrose preference rate while miR-16 negatively correlated with sucrose preference rate of the sucrose consumption test. Our study suggests that MD and CUPS induced different depression-like behaviors in rats. Depression induced by MD but not CUPS was significantly associated with upregulation of miR-16 and possibly subsequent downregulation of BDNF in hippocampus.

## Introduction

Depression is a highly complicated psychiatric disorder that can greatly damage the social functions of the sufferers. This disorder has become a major burden for our society [1]. Raising the monoamine level in synaptic clefts has been a major strategy for treating depression during the past 50 years. However, 40 to 50 percent of patients with depression show little or no response to this treatment [2], suggesting that monoamine depletion might not be the only pathogenetic process of depression.

Brain-derived neurotropic factor (BDNF) is the most abundant neurotrophin in the mammalian central nervous system [3]. Evidence from animal experiments and postmortem studies has consistently suggested that reduced BDNF level in the hippocampus is responsible for decreased proliferation of hippocampal neurons and consequent onset of depression [4]–[7]. In addition, environmental stressors could induce depressive performances by affecting hippocampal BDNF level [5], [7]–[9]. Currently, the underlying mechanisms by which environmental stressors regulate BDNF expression remain unidentified.

**Table 1:** Real time-RT-PCR primer sequence.

microRNA	primer sequence
miR-16	Forward:5'-TAGCAGCACGTAAATTGGCG-3'
	Reverse: common primer of miRNA which was applied in the kit but show no sequence
BDNF	Forward : 5'-TGGCTGACACTTTCGAACAC-3'
	Reverse: 5'-AGAAGAGGAGGCTCCAAAGG-3'
β-actin	Forward : 5'-GGAGATTACTGCCCTGGCTCCTA-3'
	Reverse : 5'-GACTCATCGTACTCCTGCTTGCTG-3'
U6	Forward: 5'-GCAAGGATGACACGCAAATTC-3'
	Reverse: The primer of U6 was applied in the kit but show no sequence

Numerous studies have implied that epigenetic mechanisms, such as microRNAs, may provide a link between environmental stressors and gene expression [10]. MicroRNAs (miRNAs) are small (∼22 bp), single-stranded noncoding RNAs that are able to regulate gene expression post-transcriptionally [11]. Many miRNAs have been identified as crucial regulators in cellular proliferation due to their ability to downregulate the expression of numerous proliferation-related genes [12], [13]. Generally, an miRNA exerts its effect through degradation of mRNA or translational repression of mRNA expression by binding to the seed sequence at the 3' untranslated region (3'UTR) [11]. Therefore, computational target analysis has been developed to predict potential regulatory miRNAs for the gene of interest [14]. Using this tool, miR-16 was identified as one of the candidate miRNAs for the BDNF gene. A recent report demonstrated that miR-16 plays an important role in the negative modulation of hippocampal neurogenesis and consequent depression-like behaviors [15]. We therefore hypothesize that: 1) Environmental stressors may induce miR-16 expression; 2) High miR-16 expression may downregulate BDNF expression; and 3) Downregulation of BDNF expression may lead to the reduction of hippocampal neurogenesis and subsequent depression-like behaviors.

**Table 2:** The comparison of behavioral indexes in open field test between groups(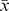
±SD).

	Total distance(cm)	Vertical activity(number)	Central area rate	Fecal pellets(number)
MD group	121.70±46.31^*△^	9.00±2.37^*^	0.07±0.03	2.17±1.47**^△^**
CUPS group	300.68±110.07^*^	9.17±2.93^*^	0.06±0.04	5.33±1.63^*^
C group	696.83±145.80	25.33±3.27	0.08±0.03	1.67±0.82
F value	43.904	63.805	0.552	12.939
P value	<0.001	<0.001	0.587	0.001

Note: *compare with C group, *p*<0.05; **^△^**compare with CUPS group, *p*<0.05;

Given the fact that the stress-induced alterations in hippocampal BDNF expression and depression-like behaviors varied with different stressors [8], [16], we tested our hypothesis in this study with two classical chronic stress paradigms: maternal deprivation (MD) and chronic unpredictable stress (CUPS). These two paradigms represent two major stressors during early life and in adulthood, respectively. We verified the role of hippocampal miR-16 and BDNF expression in early life stressor-induced depression-like behaviors.

## Materials and Methods

### 1. Ethics Statement

This study was approved by the Animal Care & Use Committee of Central South University. All experiments were performed in accordance with the Guide for Care and Use of Laboratory Animals (Chinese Council).

### 2. Animals

Sprague-Dawley rats at the age of 3 months were provided by the Animal Center of Central South University. Maximal effort was made to minimize the number of animals used. After fertilization, rats were examined at 9∶00 every day for delivery. Rat offspring born before 9∶00 were designated as postnatal day 1 (PND 1). Newborn male offspring from over 10 pregnant rats were mixed and randomly divided into three groups: maternal deprivation (MD, N = 6), chronic unpredictable stress (CUPS, N = 6) and control group (C, N = 6). Rats in MD group received maternal deprivation for 2 weeks right after birth, rats in CUPS group received chronic unpredictable stress for 3 weeks right after reaching 10-weeks old, and rats in control group received standard husbandry care.

### 3. Maternal Deprivation (MD)

The MD paradigm was conducted as previously described [17]. Briefly, rat offspring were separated from their mothers for 6 hours daily from PND 1 to PND 13 (the separations occurred between 9∶00 and 15∶00). To block communication between littermates, each offspring was placed in a single cell (each cage with dimensions of 32cm×32cm×14cm was divided into four cells of the same size) covered withhttp://dj.iciba.com/sawdust/dry sawdust. The offspring were then returned to their home cage after the experiment. All experiments were carried out in a temperature-controlled room (25°C). After PND 21, rats were housed socially with the same gender (5/cage) until adulthood (10 weeks).

**Table 3:** The comparison of behavioral indexes in forced swimming test and sucrose consumption test between groups (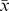
±SD).

	Rest floating time (s)	Sucrose preference rate
MD group	145.67±55.24^*△^	0.42±0.125^*△^
CUPS group	236.00±28.88^*^	0.55±0.10^*^
C group	92.00±31.45	0.77±0.05
F value	19.556	20.535
P value	<0.001	<0.001

Note: *compare with C group, *p*<0.05; **^△^**compare with CUPS group, *p*<0.05;

### 4. Chronic Unpredictable Stress (CUPS)

The CUPS paradigm was performed following a previously an established protocol [18] with minor modifications. Briefly, rats at 10-weeks old were randomly exposed to one of the following stressors once a day for 3 weeks: water deprivation for 24 hrs, food deprivation for 24 hrs, swimming in cold water (4°C) for 5 min, exposure to an elevated open platform (10 cm×10 cm, 160 cm in height) for 2 hrs [19], restraint stress for 2 hrs [20] or electric foot shock for 20 s (800 mA, 1-s duration, average 1 shock/10 s). Stress was given at different times of the day to establish unpredictability.

**Figure 1:**
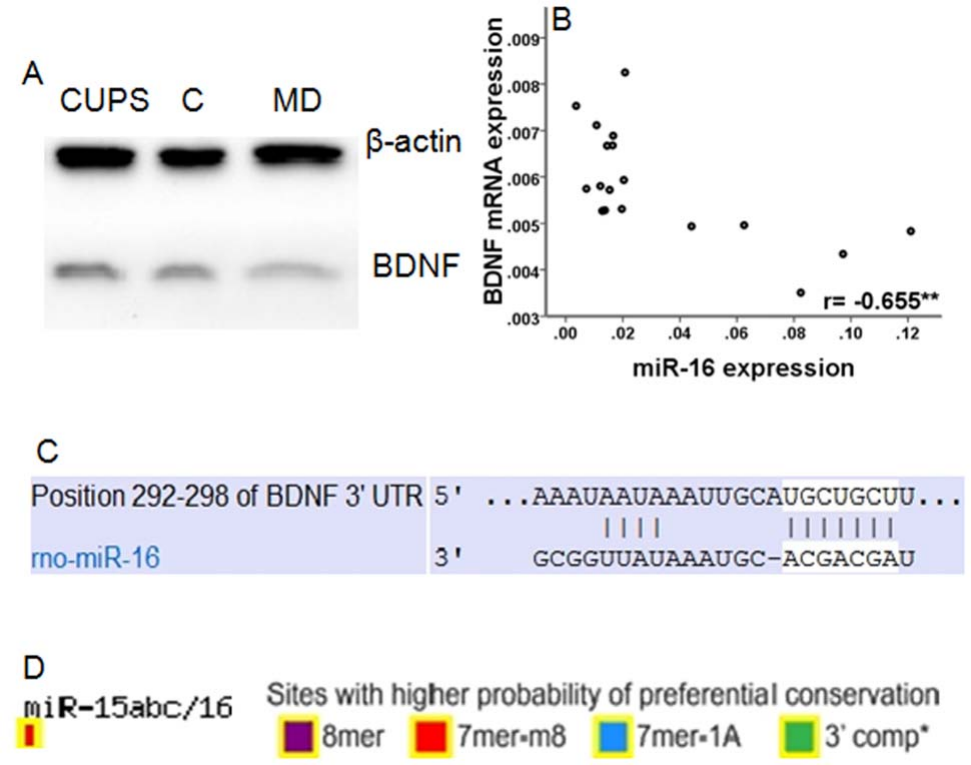
The relationship between miR-16 and BDNF. D) Western blot of BDNF protein levels. B) Correlation between BDNF mRNA expression and miR-16 expression. ^**^ *p*<0.01. C) The proposed binding site of miR16 at the 3'UTR of BDNF. D) The evolutionary conservation of the mir-16 binding site in BDNF.

**Table 4:** The comparison of BDNF and miR-16 mRNA expression between groups(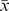
±SD).

	BDNF mRNA	BDNF protein	miR-16
MD group	0.0046±0.0006^*△^	0.3117±0.0649^*△^	0.0711±0.0367^*△^
CUPS group	0.0063±0.0012	0.6500±0.2802	0.0151±0.0043
C group	0.0065±0.0007	0.7450±0.3602	0.0123±0.0055
F value	8.266	4.396	14.184
P value	0.004	0.031	<0.001

Note: *compare with C group, *p*<0.01; **^△^**compare with CUPS group, *p*<0.01;

### 5. Open Field Test

Open field test was carried out as previously described [21]. The open field arena was made of an open rectangular plastic box (100 cm×100 cm×30 cm) with 25 squares (20 cm×20 cm) painted on the floor. The 25 squares included 16 peripheral squares and 9 central squares. At the time of test, rats were placed individually at the center of the field and were allowed to explore the area freely for 5 min. The activity of the rats was recorded by an overhanging camera that was linked to a computer. Ethovision 3.0 (Noldus, The Netherlands) was used to track the behaviors of the rats. The total distance a rat moved in the arena, distance a rat moved in the central squares, the number of vertical activity, and the number of fecal pellets present in the arena during the 5 min test were recorded. The arena was cleaned with 70% alcohol between trials to make sure a rat's behaviors were not affected by the imprint of previous rats. All behavioral tests were carried out when rats reached 13 weeks old.

**Figure 2:**
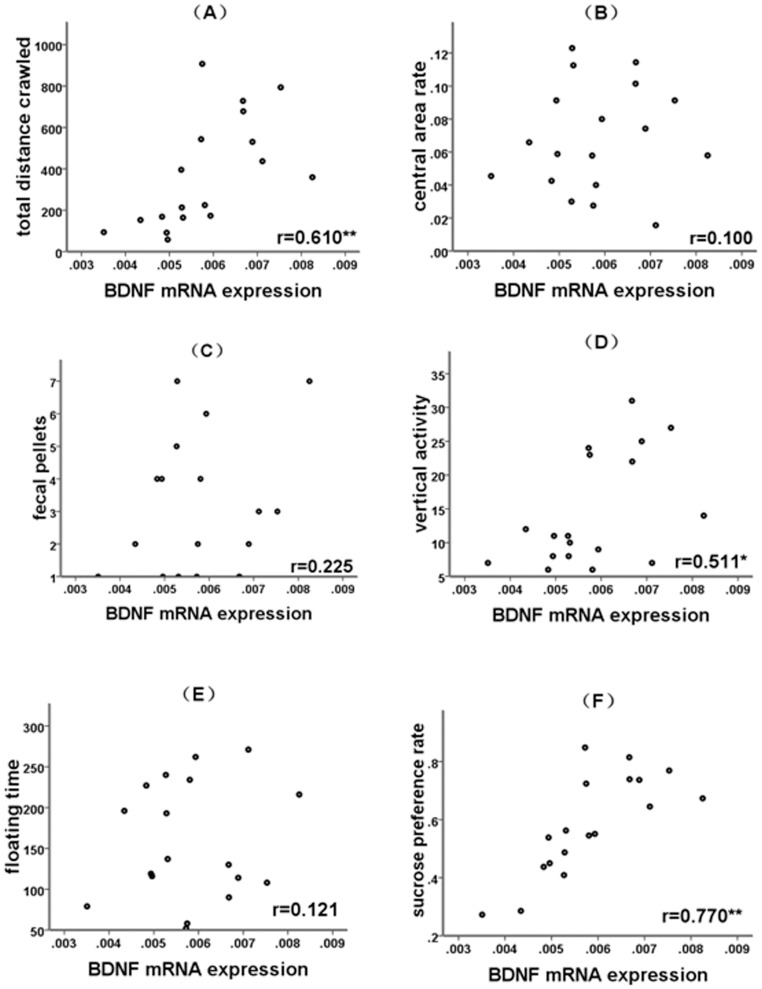
Correlation between BDNF mRNA expression and depression-like behaviors. (A) Correlation between BDNF mRNA expression and the total distance crawled. (B) Correlation between BDNF mRNA expression and the central area rate. (C) Correlation between BDNF mRNA expression and the fecal pellets. (D) Correlation between BDNF mRNA expression and the vertical activity. (E) Correlation between BDNF mRNA expression and the floating time. (F) Correlation between BDNF mRNA expression and the sucrose preference rate.^ *^ *p*<0.05,^**^ *p*<0.01.

### 6. Forced Swimming Test

Forced swimming test was performed 24 hrs after open field test following a previously established protocol [22]. Two swimming sessions were conducted: a 15-min pretest on the first day followed by a 5-min test the next day. During the test, rats were placed individually in a Pyrex cylinder (21 cm × 46 cm) filled with 25°C water to a depth of 30 cm. After swimming for 15 min on day 1, rats were dried with towels and placed back in their home cage. The water in the cylinder was emptied and refilled between rats to ensure the rat's behaviors were not affected by detecting a previous rat's scent. Twenty-four hrs after the first trial, rats were placed in the swimming apparatus again for a 5-min test trial. A video camera hung above the cylinders was used to record the rats' activity. The float time (time a rat spent in keeping its head above water with only slight movements) was recorded. The data were analyzed by two observers blind to the experimental conditions.

**Figure 3:**
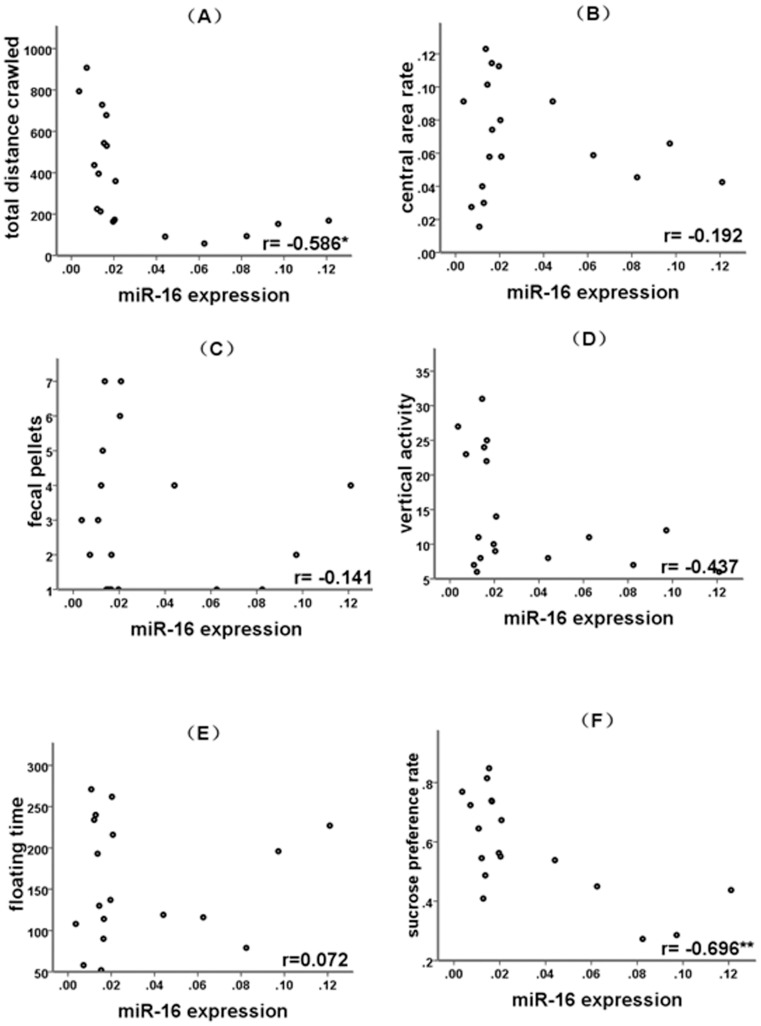
Correlation between miR-16 expression and depression-like behaviors. (A) Correlation between miR-16 expression and the total distance crawled. (B) Correlation between miR-16 expression and the central area rate. (C) Correlation between miR-16 expression and the fecal pellets. (D) Correlation between miR-16 expression and the vertical activity. (E) Correlation between miR-16 expression and the floating time. (F) Correlation between miR-16 expression and the sucrose preference rate. ^*^ *p*<0.05,^**^ *p*<0.01.

### 7. Sucrose Consumption Test

Sucrose consumption test was performed as described previously [23]. The whole test took 3 days. On day 1, rats were housed individually and given free access to two bottles of sucrose solution (1%, w/v and 100 ml). Rats were trained to adapt to sucrose solution for 24 hrs. On day 2, one bottle of sucrose solution was replaced with 100 ml of water for 24 hrs. On day 3, rats were deprived of water and food for 23 hrs, and then given free access to two pre-weighed bottles of solution: 100 ml of sucrose solution (1%, w/v) and 100 ml of water. One hr later, the consumed volume in both bottles was recorded. The sucrose preference rate was calculated using the following formula: Sucrose preference rate = sucrose consumption/(water consumption + sucrose consumption)×100%.

### 8. Sample Collection

Animals were sacrificed 24 hrs after the last behavioral test. Rats were anesthetized with intraperitoneal injections of 10% chloral hydrate (30 ml/kg body weight). Brains were rapidly removed from the skull, and hippocampus tissues were immediately sectioned according to the anatomical atlas of Paxinos and Watson [24]. Tissues were snap frozen in liquid nitrogen and kept at –80°C until use.

### 9. Real-time Reverse Transcription Quantitative PCR

Quantitative RT-PCR was used to detect BDNF mRNA and miR-16 microRNA in hippocampus tissues. Total RNA was extracted using Trizol reagent (Invitrogen, Carlsbad, CA, USA). Reverse transcription of BDNF and miR-16 was performed using RevertAid First Strand cDNA synthesis kit (MBI Fermentas, Burlington, Canada) and One Step PrimeScript® miRNA cDNA Synthesis Kit (Perfect Real Time, TaKaTa, Japan) following the manufacturer's protocol respectively. Real-time quantitative PCR was performed using Roter-Gene 3000 (Corbett Research, Sydney, Australia). The primers used for BDNF, miR-16, β-actin, and u6 are listed in Table 1. The data were analyzed by comparing miR-16 and BDNF mRNA levels with u6 and β-actin level, respectively for each rat and then subjected to statistical analysis. All qRT-PCR reactions were performed thrice.

### 10. Western Blot

Hippocampus tissues were harvested as described above. Western blots were performed as previously described [25]. The anti-BDNF (1∶1000 dilution), anti-β-actin (1∶1000 dilution), and HRP-conjugated second antibodies (1∶5000 dilution) were purchased from Santa Cruz Biotechnology (Santa Cruz, CA).

### 11. Statistical Analysis

Data was presented as mean and standard error of the mean, and the statistical package for the Social Sciences Version 17.0 was used to analyze the data. One-way analysis of variance (ANOVA) or Kruskal-Wallis LSD-test or Nemenyi-test was used for analyzing the difference between groups. Correlation between BDNF, miR-16 expression, and behavioral indexes were analyzed using Pearson correlation test. A P<0.05 was considered statistically significant.

## Results

### 1. Effect of MD and CUPS on the Behaviors of Adult Rats

Behavioral data obtained from open field test were presented in Table 2. The total distance that the rats crawled in the open field arena was significantly different between three groups (F = 43.904, *p*<0.001). MD rats crawled less distance than CUPS rats (t = –2.849, *p* = 0.012), while both MD and CUPS rats crawled less distance than control rats (*p*<0.001, *p*<0.001, respectively). The number of vertical activity was different between three groups (F = 63.805, *p*<0.001) as well. MD and CUPS rats exhibited no difference in the number of vertical activity, but their vertical activity was significantly decreased compared to control rats (*p<*0.001, *p = *0.002, respectively). No difference in central area rate was found between three groups (F = 0.552, *p* = 0.587). The number of fecal pellets was significantly different between three groups (F = 12.939, *p = *0.001). CUPS rats excreted more fecal pellets than MD and control rats (*p* = 0.001, *p*<0.001, respectively), while no difference in fecal pellets was found between MD and control rats (*p* = 0.532).

The behavioral data of forced swimming test and sucrose consumption test were presented in Table 3. Significant differences in floating time were observed between three groups (F = 19.556, *p*<0.001). CUPS and MD rats exhibited significantly longer floating times than control rats (*p<*0.001, *p* = 0.036, respectively), while CUPS rats exhibited longer floating time than MD rats (*p* = 0.001). As shown in Table 3, sucrose consumption test showed significant differences in sucrose consumption between three groups (F = 20.535, *p*<0.001). Both MD and CUPS rats consumed less sucrose than control rats (*p*<0.001, *p* = 0.001, respectively), but MD rats consumed less sucrose than CUPS rats (*p* = 0.035).

### 2. Effect of MD and CUPS on BDNF mRNA, BDNF Protein, and miR-16 mRNA Levels in the Hippocampus of Adult Rats

BDNF has about 22 transcripts. Among them, the expression of BDNF transcribed from the 5′ exon VI and 3′ common exon was analyzed in this study due to its high abundance in the hippocampus and its essential role in both neuronal survival and synaptic plasticity, which were generally considered to be involved in the development of depression [26]–[28]. As shown in Table 4, significant differences in BDNF mRNA expression were observed in the hippocampus among three groups (F = 8.266, *p* = 0.004). MD rats showed significantly lower BDNF mRNA expression than both CUPS (*p* = 0.006) and control rats (*p* = 0.002). However, no difference in BDNF mRNA expression was observed in the hippocampus between CUPS and control rats (*p* = 0.608). As shown in Table 4 and Fig. 1A, significant differences in BDNF protein expression were observed in the hippocampus among three groups (F = 4.396, *p = 0.031*). MD rats showed significantly lower BDNF protein expression than both CUPS (*p = 0.044*) and control rats (*p = 0.013*). However, no difference in BDNF protein expression was observed in the hippocampus between CUPS and control rats (*p = 0.546*). Significant differences in miR-16 expression was observed in the hippocampus of rats among three groups (F = 14.184, *p*<0.001). MD rats expressed higher levels of miR-16 than rats in both CUPS and control rats (*p*<0.001, *p*<0.001, respectively), while no difference in miR-16 expression was observed between CUPS and control rats (*p* = 0.825) (Table 4).

### 3. Correlation Analysis between BDNF mRNA and miR-16 Expression, and the Behavioral Indexes of Rats

Pearson correlation analysis revealed that BDNF mRNA expression negatively correlated with the expression of miR-16 (Fig. 1B, r = –0.655, *p* = 0.003). The computational target analysis by TargetScan (www.targetscan.net) showed that miR-16 targets the 3′UTR of BDNF with high conservation (Fig. 1C, 1D). These observations provided indirect evidence that miR-16 regulates BDNF expression. BDNF mRNA expression positively correlated with total distance rats crawled in the open field arena (Fig. 2, r = 0.610, *p* = 0.007), vertical activity (r = 0.511, *p* = 0.030) in the open field test, and the sucrose preference rate (r = 0.770, *p*<0.001) in the sucrose consumption test (Fig. 2). miR-16 expression negatively correlated with the total distance rats crawled in the open field arena (r = –0.586, *p* = 0.011) and the sucrose preference rate (r = –0.696, *p* = 0.001) in the sucrose consumption test (Fig. 3).

## Discussion

It is generally believed that various chronic environmental stressors that emerge during an individual's early development or adulthood trigger the development of depression [8], [9], [29]. However, the direct relationship between neurobiological anomalies and stress-induced depressive behaviors cannot be verified in humans due to ethical limitations. Therefore, animal models are currently the ideal tools to elucidate the biological bases of stress-induced depressive behaviors. In this study, downregulation of BDNF and upregulation of miR-16 expression were shown to be involved in MD-induced, but not CUPS-induced depressive behaviors.

Chronic unpredictable stress and maternal deprivation paradigms are commonly used to simulate the effect of adverse experiences in adulthood or early life of a person, respectively. These two paradigms are widely used to induce depressive behaviors in rodents including loss of interest, despair behaviors and anhedonia [8], [9]. In this study, CUPS and MD triggered dissimilar depressive behaviors in rats. MD induced less total distance crawled in the open field test and sucrose preference rate in sucrose consumption test than CUPS. In contrast, CUPS rats exhibited longer floating times in forced swimming test and excreted more fecal pallets in the open field test than MD rats. This suggests that negative events occurred in a person's early life led to more severe loss of interest and anhedonia than stressors that emerge in adulthood. On the contrary, stressors that emerge in adulthood induced more severe despair and anxiety-like behaviors. The existence of different depression subtypes was consistently supported by observations in animal experiments and clinics. For example, different stressors induce divergent depressive phenotypes while various depressive manifestations exist in the clinic [2]. Importantly, reduced BDNF and increased miR-16 expression was only observed in MD rats. This implies that rats suffering from CUPS in adulthood may manifest more anxious behaviors and behavioral despair from non-miR-16/BDNF mechanisms than rats in early life. On the contrary, rats experiencing MD in early life showed more severe anhedonia and loss of interest, which correlated with the upregulation of hippocampal miR-16 and dowgregulation of hippocampal BDNF expression. The changes in molecules suggest that different subtypes may be caused by different molecular pathogenesis mechanisms.

Numerous studies have demonstrated that BDNF is involved in the onset and development of depression [30], [31]. BDNF is the most widely distributed neurotrophin in the mammalian CNS with especially high levels found in the hippocampus [32]. A recent study demonstrated that MD could lead to downregulation of BDNF expression in the hippocampus [8], but the regulatory mechanisms via which MD influences BDNF expression remain unclear. In this study, BDNF expression was found to be downregulated in the hippocampus of MD rats and is negatively correlated with the miR-16 level, which is consistent with previous reports. Computational target analysis revealed that BDNF is a target gene of miR-16. Given the evidence that miRNAs provide a link between environmental factors and gene expression [10] and high correlation between BDNF and miR-16 expression in the hippocampus (p = 0.003), we proposed that miR-16 regulates BDNF expression in MD rats. However, several other targets of miR-16, such as 5-HT transporter and BCL-2 [15], [33] genes, have also been reported to be involved in hippocampal neurogenesis and correspond to depressive state. It is reasonable to believe that miR-16 may play a crucial role in the development of depression via simultaneously modulating the expression of multiple genes with different physiological functions, such as BDNF’s role in neurogenesis and BCL-2′s involvement in neuron survival and apoptosis. The involvement of other miR-16-targeted genes in MD-induced depression needs further validation. However, why CUPS-induced depressive behaviors exhibited no association with BDNF and miR-16 expression is unclear.

In this study, upregulation of miR-16 and downregulation of BDNF mRNA expression in hippocampus were only observed in MD-treated rats, but not in CUPS rats. This suggests that different stressors-induced divergent depressive phenotypes may result from dissimilar molecular underpinnings. This divergence was also reported recently. For example, stress-induced increase or no change in BDNF level in animal models of depression was observed in two studies [9], [16]. The discrepancy in BDNF expression may result from various factors. Among them, the different types of stressors used for inducing depression are catching more and more attention recently [8], [16]. BDNF has been considered as a molecular marker for neuronal plasticity due to its crucial role in neuronal proliferation, neurite growth and remodeling, and synapse formation [7]. Reduced hippocampal BDNF expression was only observed in MD-treated rats in this study. This suggested that early life stress might damage the hippocampal neuronal plasticity of the sufferers, and it might be the basis of the subsequent depression-like behavior shown in adulthood. In addition,BDNF reduction-mediated decrease in neuronal plasticity is sustainable, and the depression-related neurobiological anomaly may be hard to be reshaped to normal state. This can explain why MD-induced depressive behaviors do not disappear after the animals were reared in normal environment for 8 weeks in this study. In contrast, CUPS-induced behavioral anomaly in adult rats usually disappeared after 6 weeks of housing in a normal environment [34].

In conclusion, results from this study suggest that different psychological stressors, such as maternal deprivation and chronic unpredictable stress, induce dissimilar depressive performances in rats, among which the MD-induced depression-like behaviors may be associated with the upregulation of miR-16 and subsequent downregulation of BNDF in the hippocampus.
